# 

**Published:** 1868-01

**Authors:** 


					﻿VOL. XXII.	No. 1.
THE
Rental llcgi.Gtcr.
... z
EDITED BY
<- , ■ , ;
J. TAFT & G. WATT.
JANUARY, 1868.	?
MONTHLY:
THREE DOLLARS PER ANNUM, IN ADVANCE.
CINCINNATI:
WRIGHTSON &. CO-, Printers, 167 WALNUT STREET.
«7. <* WJf, TAFT, Dmtixtx, 2.‘{S Wost Fourth St.
				

